# Chicago Marine Hospital

**Published:** 1849-07

**Authors:** 


### ARTICLE III.

### CHICAGO MARINE HOSPITAL.

This institution now in process of erection, is to be situated on the point between the river and the lake, being the ground occupied as a military post, and will overlook the harbor so as to produce a beautiful effect. The building will front West on Michigan avenue, and will be convenient to all the marines of the city.

The building will be 90 feet 6 inches front by 128 feet 6 inches deep, three stories high, above a basement, with a cupola elevated to 67 feet.

The appearance will be fine, and the manner of its construction permanent. The basement will be of stone, the other walls of brick.

It will contain forty-eight apartments, besides water closets bath rooms, &c., and will accommodate three hundred an[illegible] fifty patients.

Thus, under the auspices of the general government, ha[illegible] been commenced a noble charity, whose blessings will be fe[illegible] and appreciated by the homeless mariner, through a lon[illegible] succession of ages. E.

